# Thin Films of a Complex Polymer Compound for the Inhibition of Iron Alloy Corrosion in a H_3_PO_4_ Solution

**DOI:** 10.3390/polym15214280

**Published:** 2023-10-31

**Authors:** Yaroslav G. Avdeev, Tatyana A. Nenasheva, Andrei Yu. Luchkin, Andrei I. Marshakov, Yurii I. Kuznetsov

**Affiliations:** A.N. Frumkin Institute of Physical Chemistry and Electrochemistry, Russian Academy of Sciences, 31 Leninskii Prospect, 119071 Moscow, Russia; avdeevavdeev@mail.ru (Y.G.A.); tnenasheva@inbox.ru (T.A.N.); mar@ipc.rssi.ru (A.I.M.); yukuzn@gmail.com (Y.I.K.)

**Keywords:** acid corrosion, polymer film, corrosion inhibition, coordination polymer compounds, triazole, hydrogen permeation into the alloys, iron alloys, phosphoric acid

## Abstract

The etching of iron alloy items in a H_3_PO_4_ solution is used in various human activities (gas and oil production, metalworking, transport, utilities, etc.). The etching of iron alloys is associated with significant material losses due to their corrosion. It has been found that an efficient way to prevent the corrosion of iron alloys in a H_3_PO_4_ solution involves the formation of thin complex compound films consisting of the corrosion inhibitor molecules of a triazole derivative (TrzD) on their surface. It has been shown that the protection of iron alloys with a mixture of TrzD + KNCS in a H_3_PO_4_ solution is accompanied by the formation of a thin film of coordination polymer compounds thicker than 4 nm consisting of TrzD molecules, Fe^2+^ cations and NCS^−^. The layer of the complex compound immediately adjacent to the iron alloy surface is chemisorbed on it. The efficiency of this composition as an inhibitor of iron alloy corrosion and hydrogen bulk sorption by iron alloys is determined by its ability to form a coordination polymer compound layer, as experimentally confirmed by electrochemical, AFM and XPS data. The efficiency values of inhibitor compositions 5 mM TrzD + 0.5 mM KNCS and 5 mM TrzD + 0.5 mM KNCS + 200 mM C_6_H_12_N_4_ at a temperature of 20 ± 1 °C are 97% and 98%, respectively. The kinetic parameters of the limiting processes of hydrogen evolution and permeation into an iron alloy in a H_3_PO_4_ solution were determined. A significant decrease in both the reaction rate of hydrogen evolution and the rate of hydrogen permeation into the iron alloy by the TrzD and its mixtures in question was noted. The inhibitor compositions 5 mM TrzD + 0.5 mM KNCS and 5 mM TrzD + 0.5 mM KNCS + 200 mM C_6_H_12_N_4_ decreased the total hydrogen concentration in the iron alloy up to 9.3- and 11-fold, respectively. The preservation of the iron alloy plasticity in the corrosive environment containing the inhibitor under study was determined by a decrease in the hydrogen content in the alloy bulk.

## 1. Introduction

Solutions of HCl and H_2_SO_4_ are widely used to clean iron alloy structures and products from various mineral contaminants, primarily thermal scale and rust [1,2]. During these operations, corrosion inhibitors (CorIs) are added to the aqueous environments in order to protect iron alloys from the corrosive effects of acid solutions [3,4,5,6,7,8]. Solutions of phosphoric acid can be an alternative to the industrial use of HCl and H_2_SO_4_ solutions for the removal of mineral contaminants from iron alloy surfaces. The use of H_3_PO_4_ in these operations in comparison with HCl and H_2_SO_4_ is advantageous due to the high rate of dissolution of iron oxides in this environment [9,10], which provides rapid removal of thermal scale and rust from the iron alloy surface.

The most interesting acid CorIs of iron alloys are compounds capable of forming protective polymer films of organic compounds on the metal surface. Low-molecular-weight unsaturated organic compounds can serve as such inhibitors. As they are adsorbed on the catalytic surface of a corroding steel, they undergo polymerization to form an organic polymer layer. The latter blocks the metal from the aggressive environment, preventing iron alloy corrosion in acid environments, often under high-temperature corrosion conditions (above 80 °C) [11].

The range of inhibitors recommended for the protection of iron alloys in H_3_PO_4_ includes quaternary ammonium salts [12], triphenylmethane derivatives [13,14], nitrogen-containing heterocycles [15] and surfactants [16]. Most often, these compounds are used in the form of composite inhibitors with halide anions [13], thiocyanate anions [14] and soluble cerium compounds [16] (Table 1). These combinations make it possible to provide the most effective protection of iron alloys in H_3_PO_4_ and reduce the consumption of substances used to this end. For example, the possibility of protecting mild steel in 1 M H_3_PO_4_ (40 °C) by adding a mixture of methyl violet and NaBr is considered in [13]. The degree of protection of steel by the addition of 1 mM methyl violet is 41.2%, and by the addition of 50 mM NaBr, it is 31.1%. The degree of protection of steel by a mixture of 1 mM methyl violet and 50 mM bromide is 91.8%.

Compounds used to protect iron alloys from corrosion in solutions should not only slow down the overall corrosion of iron alloys but also prevent the penetration of hydrogen, which can lead to brittleness and the deterioration of mechanical properties, into the metal [17,18,19,20,21]. Information on the effect of inhibitors on the bulk sorption of hydrogen by iron alloys during corrosion in solutions of hydrochloric and sulfuric acids is reported in only a few works [22,23,24,25]. We failed to find information about the effect of CorIs on the bulk sorption of hydrogen by iron alloys in H_3_PO_4_ solutions.

It seems important to assess the effect of CorIs on the bulk sorption of hydrogen by iron alloys and its corrosion in a H_3_PO_4_ solution. We studied 1,2,4-triazole derivative (TrzD, [(C_6_H_5_CH_2_)_3_N–C_2_H_2_N_3_]Cl) as a CorI. Compounds of the triazole group are being actively studied as CorIs for iron alloys in acidic environments and show high efficiency [26]. To enhance the protective effect of TrzD against iron alloy corrosion in phosphoric acid solutions, we added potassium thiocyanate and hexamethylenetetramine. Potassium thiocyanate is widely used to enhance the protection of iron alloys in acid environments by nitrogen-containing CorIs [14,27,28,29,30]. Hexamethylenetetramine [31,32,33,34,35,36,37], which by itself weakly slows down the corrosion of iron alloys, is also often used as a component of mixed inhibitors. We hypothesized that mixed inhibitors based on TrzD are capable of forming polymer protective layers on the steel surface, which would provide not only efficient inhibition of the corrosion process of iron alloys in H_3_PO_4_ but also prevent the bulk sorption of evolved hydrogen.

## 2. Materials and Methods

### 2.1. Materials

Low-carbon steel (LS, wt.: 0.05% carbon, 0.05% chromium, 0.38% manganese, 0.15% copper, 0.09% nickel, 0.16% aluminum, 0.03% silicon, 0.04% sulfur, 0.035% phosphorus) and high-strength steel (HS, wt.: 0.7% carbon, 0.52% manganese, 0.3% chromium, 1.52% silicon) were studied as iron alloys (Norilsk, RF). The studies were carried out in H_3_PO_4_ aqueous solutions, which were de-aerated with Ar gas. The H_3_PO_4_ solutions were prepared from distilled water and 87% H_3_PO_4_ (“chemically pure” grade, Dzerzhinsk RF). TrzD, which is a chemically modified triazole, was used as the inhibitor. TrzD was added to the etching solutions as a concentrated solution in ethyl alcohol; thus, the concentration of ethyl alcohol in the acid solution was 0.25 M. Also, the addition of KNCS (“chemically pure” grade, Dzerzhinsk RF) and C_6_H_12_N_4_ (“chemically pure” grade, RF) was used.

The basic content of individual TrzD, KNCS and C_6_H_12_N_4_ in the corrosion system is 5.0, 0.5 and 200 mM, respectively. When these compounds are added as inhibitor compositions, the contents of TrzD, KNCS and C_6_H_12_N_4_ are 5.0, 0.5 and 200 mM, respectively.

### 2.2. Methods

#### 2.2.1. The Electrochemical Hydrogen Desorption Method

**Figure 1:**
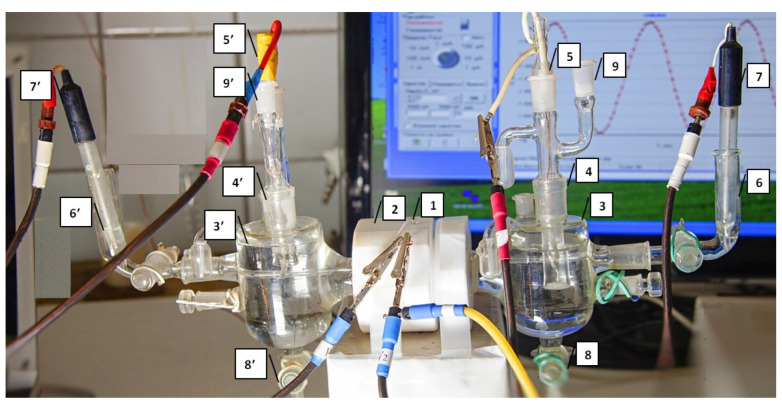
Glass cell. 1—membrane; 2—fasteners; 3—working side; 3’—diffusion side; 4, 4’—cell Pt of the electrode; 5, 5’—Pt electrode; 6, 6’—cell of the silver chloride electrode; 7, 7’—silver chloride electrode; 8, 8’—tap for draining the solution; 9, 9’—solution input into the cell.

The rate of hydrogen incorporation into the metal was determined in Devanathan–Stachurski cells (Figure 1) [38,39]. The working electrode (membrane) was made of LS (area 4.25 cm^2^, thickness 0.1 mm). One side of the membrane (diffusion) was coated with a layer of palladium. A detailed description of the preparation and the experiment itself is given in [24].

#### 2.2.2. Method IPZ Analysis

Hydrogen evolution reaction (HER) on iron in acidic solutions occurs according to the “discharge–chemical recombination, mixed rate control” or to the “slow discharge–irreversible chemical recombination” mechanisms [40,41]. If hydrogen ions are discharged on a metal surface occupied by H atoms and the inhibitor particles, the cathodic current density (*i*_c_) is expressed by [42]

*i*_c_ = *Fk*_c_
*a*_H+_ [(1 − *θ*_Inh_)*^r^*^1^ − *θ*_H_] exp(−α*FE*/*RT*), (1)

where *k*_c_ is the rate constant of discharge of H^+^ ions, *a*_H+_ is the activity of H^+^ ions, *θ*_H_ and *θ*_Inh_ are the surface hydrogen coverage and surface coverage with CorI particles, respectively, *r*_1_ is the number of hydrogen adsorption sites occupied by inhibitor particles, α is the transfer coefficient, obtained by the cathodic polarized curve, *E* is the applied potential, *F* is the Faraday constant, *R* is the gas constant, and *T* is the temperature.

The kinetic parameters of HER can be obtained by IPZ analysis [40]. Here, we will only give the equations required for the calculation of these parameters. 

The rate of molization of hydrogen atoms (recombination current density, *i_r_*) can be expressed as

*i*_r_ = *i*_c_ − *i*_p_ = *Fk*_*r*_*θ*_H_^2^ (2)

where *i*_p_ is current density corresponding to the rate of hydrogen penetration through the membrane, and *k_r_* is the rate constant of hydrogen molization. 

The steady-state current density *i*_p_ can be expressed by the following equations:

*i*_p_ = *F*(*k*_abs_*θ*_H_ − *k*_*des*_*C*^*s*^_H_) (3)

and

(4) $$i_{p}=\frac{FDC_{H}^{s}}{L}$$

where *k*_abs_ and *k*_des_ are the rate constants of surface sorption and desorption of *H* atoms from the metal, *C_H_^s^* is the surface hydrogen concentration, *L* is the membrane thickness, and *D* is the diffusion coefficient.

It follows from Equations (3) and (4) that

(5) $$\theta_{H}=\frac{k_{des}+\frac{D}{L}}{k_{abs}}C_{H}^{s}=kC_{H}^{s}$$

where *k* is the kinetic diffusion constant that shows the ratio of the concentrations of hydrogen atoms on the surface and in the metal phase.

Using Equations (1), (2), (4) and (5), the following equations can be obtained [24,40]:

(6) $$i_{c}\exp\left(\frac{\alpha FE}{RT}\right)=Fk_{1}a_{H^{+}}{\left(1-\theta_{inh}\right)}^{r1}-\frac{k_{1}a_{H^{+}}kL}{D}\cdot i_{p}$$

(7) $$i_{p}=\frac{D\sqrt{F}}{Lk\sqrt{k_{r}}}\cdot \sqrt{i_{c}-i_{p}}=\frac{D\sqrt{F}}{Lk\sqrt{k_{r}}}\sqrt{i_{r}}$$

By combining Equations (1), (2), (4) and (5), the expression for calculating the surface hydrogen coverage can be obtained:

(8) $$\theta_{H}=\frac{-\left(k_{1,i}+\frac{D}{Lk}\right)+\sqrt{{\left(k_{1,i}+\frac{D}{Lk}\right)}^{2}+4k_{r}k_{1,i}{\left(1-\theta_{inh}\right)}^{r1}}}{2k_{r}}$$

where $k_{1,i}=k_{1}a_{H^{+}}\cdot \exp\left(\frac{\alpha FE_{i}}{RT}\right)$ is the formal rate constant of hydrogen ion discharge reaction at the *E_i_* potential. The surface concentration of diffusion-mobile hydrogen in the metal $\left(C_{H}^{s}\right)$ can be calculated from *i*_p_ values using Equation (4) or from *θ_H_* values using Equation (5).

#### 2.2.3. Electrochemical Impedance Spectroscopy (EIS)

Thermodynamic aspects of sorption of TrzD on the LS surface were studied using the capabilities of the EIS method. A cathodic polarized (*E* = −0.30 V) rotating disk (LS, S = 0.64 cm^2^, *n* = 1000 rpm) was used as a working electrode during the experiment. An LS electrode was placed in the aggressive environment and kept until a stationary impedance spectrum was obtained (2 h); then, the required concentration of the Cor*I* (*C_inh_*) was added to the aggressive environment, and the electrode was kept until a stationary spectrum (3 h) was obtained. Electrochemical impedance of the *LS* electrode was measured in the frequency range of 10 mHz–3 kHz with an alternating voltage amplitude of 0.020 V.

The *LS* coverage with the inhibitor (*θ_inh_*) was calculated using the following expression:

(9) $$\theta_{inh}=\frac{C_{\mathrm{dl}}^{0}-C_{\mathrm{dl}}}{C_{\mathrm{dl}}^{0}-C_{\mathrm{dl}}’},$$

where *C*^0^*_dl_*, *C_dl_* and *C_dl_*’ are the capacitance of the double electric layer of the LS in the medium without the addition of Cor*I*, in the inhibited medium and under conditions of the limiting CorI sorption on the electrode surface.

#### 2.2.4. Gravimetric Method

The corrosion rate of HS was calculated based on the change in the mass of the metal coupon (85 mm × 10 mm × 0.5 mm) in 2 M H_3_PO_4_:

(10) $$\rho =\frac{\Delta m}{S\tau }100\%$$

where Δ*m* is the mass loss of the metal coupon mass, g; *S* is the metal coupon area, m^2^; and *τ* is the duration of the experiment, h. These studies were carried out in static environments with free access to air. Additionally, the linear corrosion rate was calculated:

(11) $$CR=\frac{8.76\rho }{d}$$

where *d* is the density of the metal, g/cm^3^.

The inhibitory factor, which quantitatively characterizes the effect of additives on the corrosion rate, is described as

(12) $$Z=\frac{\rho_{0}-\rho_{inh}}{\rho_{0}}100\%$$

where *ρ_inh_* and *ρ*_0_ are the corrosion rates of metal coupons in an inhibited medium and in the same medium without a Cor*I*.

#### 2.2.5. Vacuum Extraction Method

The hydrogen content in the volume of the alloy was determined using the vacuum extraction method on samples from HS after soaking in a corrosive environment. Before starting the experiment, air was pumped out of the vessel (residual pressure in the vessel 1.33 × 10^−4^ Pa). The samples were placed in a vessel and heated to 500 °C. Measurements of hydrogen absorbed volume by the metal were carried out for 10 min by changing the pressure (*P_total_*) using a McLeod pressure gauge. Then, the pressure of released *H* (*P_H2_*) was calculated using the following equation:

*P*_*H*2_ = *P*_*total*_ − *P*_*correct*_, (13)

where *P_correct_.* is the correction for the blank test. 

The number of *H* atoms in the metal was determined by the following equation:

(14) $$C_{H}^{v}=\frac{KP_{H2}}{V}$$

*K* is the correction factor, taking into account atmospheric pressure and the volume of the material.

The calculation $C_{H}^{v}$ took into account the amount of metallurgical hydrogen (2.4 × 10^−6^ mol/mL).

The inhibition coefficient for the introduction of hydrogen into the volume of the alloy was calculated using the following equation:

(15) $$Z_{H}^{v}=\frac{C_{H}^{v}-C_{H,inh}^{v}}{C_{H}^{v}}100$$

where *C^v^_H_* and *C^v^_H,inh_* are the volumetric concentration of *H* atoms in the background and the solution containing Cor*I*, respectively.

#### 2.2.6. Determination of Alloy Ductility

To study the mechanical properties of the alloy, an NG-1-3M installation was used. The number of kinks before the destruction of the material without (*β*_0_) and after exposure to an acid solution (*β*) was determined. The ductility of the iron alloy was then calculated using the following expression:

*p* = *ββ*_0_^−1^ 100% (16)

The mean value for the HS studied was *β*_0_ = 87.

#### 2.2.7. XPS Measurements of LS Surfaces

The composition of the surface layers formed by the TrzD inhibitor on LS was studied using X-ray photoelectron spectroscopy (XPS) on an HB100 Auger microscope (VG, London, UK). The design of the microscope was supplemented with a camera for recording XPS spectra. Round coupons (LS, 10 mm in diameter) served as the samples in the XPS measurements. The coupons were pretreated in the same way as in the corrosion studies.

The binding energy of electrons (*E*_b_) knocked out from the internal shells of atoms was calibrated with respect to the XPS peak of C1s electrons from the vapors of the deposited layers of diffusion oil. The binding energy of C1s electrons was 285.0 eV. The peaks of the following chemical elements were obtained: Fe2*p*, Fe3*p*, O1*s*, C1*s*, N1*s*, S2*p* and P2*p*. Quantitative characteristics based on the photoionization cross sections of the corresponding electron shells were published by Wagner [43]. The integral peak intensities were obtained after background subtraction and by fitting the observed peaks by Gaussian curves [44].

Ultrasonic cleaning of metal coupons in an aqueous environment (20 min) was carried out to determine the nature of the connection between CorI molecules and the LS surface. This cleaning removes the inhibitor molecule from the sample surface that is bound to it by physical forces. This procedure does not remove chemically bound CorI molecules from the surface of the LS coupons.

#### 2.2.8. Atomic Force Microscopy

The surface topography was measured in open air using a SolverNext II atomic force microscope manufactured by NovaPhotonix LLC (Sankt-Peterburg, Russia) in amplitude modulation mode. A silicon probe with a conductive platinum coating, a resonance frequency of 73 kHz, and an elasticity coefficient of 4.5 N/m was used.

#### 2.2.9. Kelvin Probe Force Microscopy

Surface potential (VCPD) of the surface was measured by two-pass Kelvin probe force microscopy (KZSM) in the amplitude modulation mode on an atomic force microscope SolverNext II manufactured by NovaPhotonix LLC (RF) under open atmosphere conditions. A silicon probe with a conductive platinum coating, a resonance frequency of 73 kHz and an elasticity coefficient of 4.5 N/m was used. The height of the second pass was 10 Nm. Prior to measurements, the probe was calibrated on a fresh surface of highly oriented pyrolytic graphite (HOPG). 

Electron yield work (VCPD) was calculated considering the electron yield work of the probe material (WTIP = 4.8 V) VCPD = (WTIP − WSAMPLE)/e, where WSAMPLE is the electron yield work of the sample material, and e is the elementary electric charge.

All experimental data presented in the article refer to temperature (*t* = 20 ± 1 °C). Current–voltage and EIS studies were performed using a potentiostat manufactured by Kronas LLC (Moscow, Russia). Electrochemical measurements were carried out using silver-silver chloride reference electrodes and platinum counter electrodes. All potentials are given vs. the standard hydrogen electrode.

## 3. Results and Discussion

### 3.1. Current–Voltage Curves

**Figure 2:**
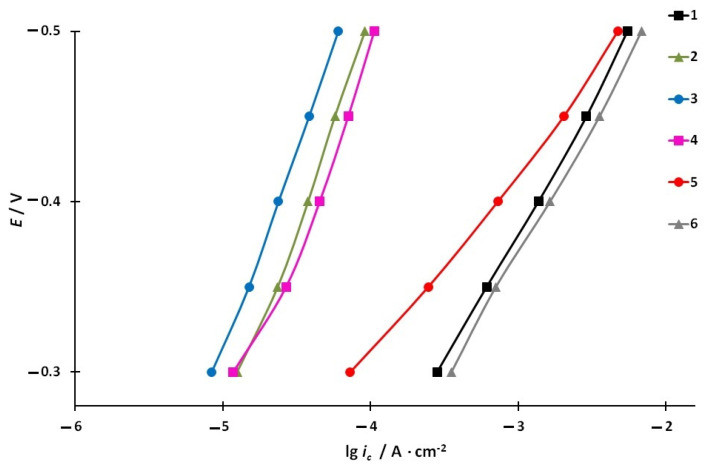
The dependence of cathode current density on potential on LS in 2M H_3_PO_4_ (1) containing TrzD (2), TrzD + KNCS (3), TrzD + KNCS + C_6_H_12_N_4_ (4), KNCS (5) and C_6_H_12_N_4_ (6). *t* = 20 ± 1 °C.

**Figure 3:**
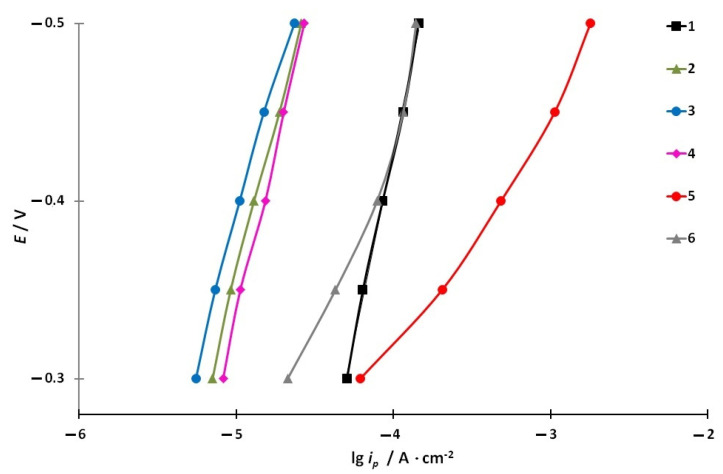
The dependence of the hydrogen insertion current density on the potential of an LS membrane in 2M H_3_PO_4_ (1) containing TrzD (2), TrzD + KNCS (3), TrzD + KNCS + C_6_H_12_N_4_ (4), KNCS (5) and C_6_H_12_N_4_ (6). *t* = 20 ± 1 °C.

**Figure 4:**
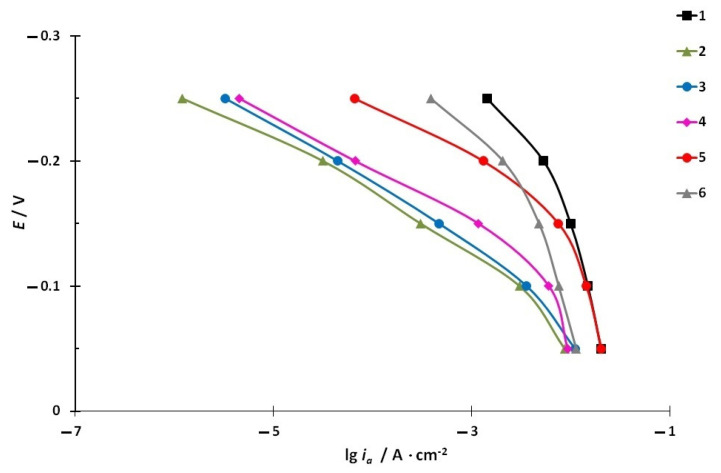
The dependence of anode current density on potential on LS in 2M H_3_PO_4_ (1) containing TrzD (2), TrzD + KNCS (3), TrzD + KNCS + C_6_H_12_N_4_ (4), KNCS (5) and C_6_H_12_N_4_ (6). *t* = 20 ± 1 °C.

Cathodic and anodic current–voltage curves and the dependence of *i*_p_ on the potential of phosphoric acid with various additives were obtained (Figure 2, Figure 3 and Figure 4). As one can see, in the presence of TrzD, both in pure form and in a mixture, the cathodic and anodic currents and the rate of hydrogen penetration into the metal decrease significantly.

The addition of hexamethylenetetramine to the acid solution slightly reduces the rate of the anodic process without significantly affecting the evolution and penetration of hydrogen into the iron alloy. An addition of 2M H_3_PO_4_ thiocyanate leads to an increase in the penetration of H atoms into the iron alloy. Quantitative data on the effect of the studied additives on the corrosion potential of LS in 2 M H_3_PO_4_ and cathodic and anodic reactions of LS are given in Table 2.

### 3.2. Determination of the Surface Coverage with CorI Particles

**Figure 5:**
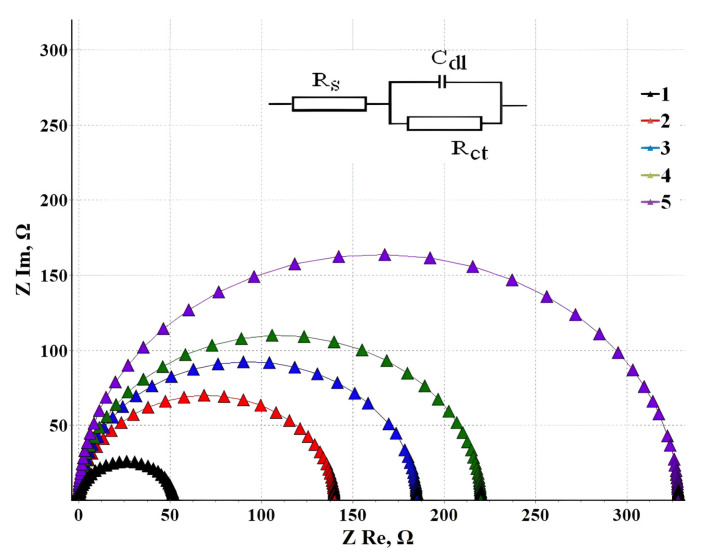
Reaction scheme and Nyquist dependence on LS in 2M H_3_PO_4_ (1) taken after addition of 5.0 µM TrzD (2, 3) and 5.0 µM TrzD + 0.5 mM KNCS to the solution (4, 5) at the following exposure times (min): 2, 4–30 and 3, 5–60. *t* = 20 ± 1 °C.

To determine *θ*_inh_ in 2M H_3_PO_4_ solutions containing corrosion inhibitors (CorIs), the EIS method was used. In the 2M H_3_PO_4_ solution, both with and in the presence of TrzD or TrzD + KNCS, the Nyquist plots of the HS electrode represent a semicircle. The structure of an electrical double layer (EL) can be formally represented as an electrical circuit (Figure 5). It includes the following elements (resistances *R_s_*, *Rct* and capacitor), arranged in a series-parallel circuit. In our case, R_S_ is the resistance of the solution, *R_ct_* is the impedance, which characterizes the maximum speed of the electrochemical reaction of H^+^ reduction on the LS, and *C_dl_* is the conditionally independent capacitance determined by the capacitance of the EL on the LS.

The radius of the Nyquist curves of LS in the studied systems in the presence of CorIs increases with longer exposure of the metal to a corrosive environment, which is most likely for the slow sorption of the studied compounds on the surface. With the same duration of the experiment, the size of the Nyquist curves obtained for LS in a solution of H_3_PO_4_ with the addition of TrzD is smaller than in a solution containing TrzD and 0.5 mmol L^−1^ KNCS.

**Figure 6:**
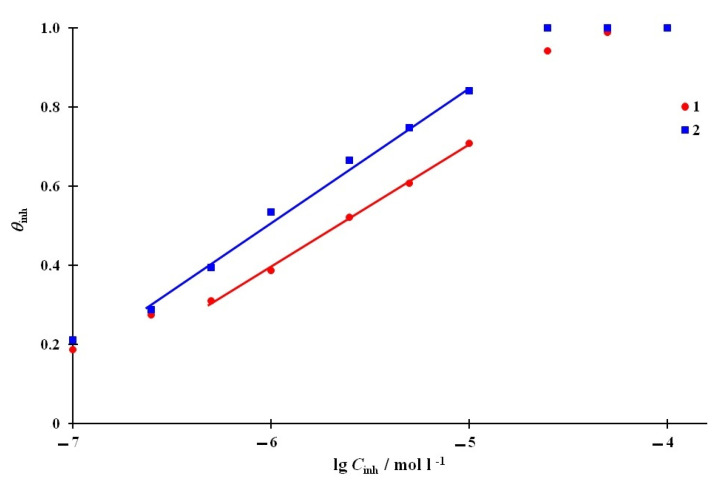
Adsorption isotherm of Cor*I* in 2M H_3_PO_4_ on LS: TrzD (1) and TrzD + KNCS (2). Theoretically calculated data (straight line), obtained during the experiment (points). *t* = 20 ± 1 °C.

The stationary *θ*_inh_ values calculated using Equation (9) were 0.9–0.99. We used these values to calculate the surface and volume sorption of hydrogen released during the cathodic reaction. Since the adsorption of TrzD on the HS develops with time, we calculated the value of *θ_inh_* using the stationary *C*_dl_ values of the electrode that were established in 2 h. The plot of the surface LS coverage with Cor*I* particles on its content TrzD in the H_3_PO_4_ solution (adsorption isotherm) is presented in Figure 6.

### 3.3. Calculation of Surface and Volume Sorption of Hydrogen Released during the Cathodic Reaction

To assess the effect of surfactants on the amount of bulk hydrogen sorbed by the metal, it was necessary to determine the surface coverage of the metal with hydrogen and the surface concentration of diffusion-mobile hydrogen in the LS. This makes it possible to perform IPZ analysis both in the background solution and in solutions containing various additives (see Section 2.2.2).

Using the experimental data shown in Figure 2 and Figure 3, namely, the dependence of the current–voltage curves and the hydrogen insertion current density on the potential along with the value of *θ*_inh_, in accordance with the IPZ analysis procedure (Equations (6) and (7)), the following values were calculated: the reaction rate constants for the discharge of hydrogen ions at potential *E*_i_ (*k*_1,i_), the rate of molization of hydrogen atoms (*k*_r_) and kinetic-diffusion constants (*k*) in a 2M H_3_PO_4_ solution containing various additives (Table 3). The constant *k* characterizes the relationship between hydrogen atoms located on the surface and in the bulk of the LS.

The average values *θ***_H_** for solutions of various compositions calculated using Equations (1) and (8) are given in Table 3.

The amount of bulk hydrogen in LS ($C_{H}^{s}$) was calculated using Equations (4) and (5), and average values are given (Table 3).

As can be seen, when TrzD is added to an acid solution, both individually and in mixtures, the hydrogen evolution reaction constants decrease, and the kinetic-diffusion constants (*k*) increase. Which leads to a decrease in the amount of both surface hydrogen (*θ*_H_) and hydrogen in the bulk of the metal ($C_{H}^{s}$) (Table 3). It was found that the maximum effect is observed in a mixture of triazole with thiocyanate (TrsD + KNCS). Compared to a pure acid solution, in a solution containing CorI, the amount of hydrogen in the alloy decreases 10 times.

It can be expected that the addition of triazole to a 2 M H_3_PO_4_ solution, even in small quantities, will affect the mechanical properties of the material and reduce the susceptibility of the steel to corrosion cracking. This is especially important for structures that are operated under load.

### 3.4. Effect of an Inhibitor on the Corrosion of LS

The corrosion of LS in a 2 M H_3_PO_4_ solution occurs at a relatively high rate of 7.8 g/(m^2^ h) (Table 4). The presence of individual TrzD in the environment studied slows down steel corrosion by a factor of 4.3. Stronger inhibition of MS corrosion is provided by mixed CorI, TrzD + KNCS and TrzD + C_6_H_12_N_4_, which reduce the corrosion rate 34- and 52-fold, respectively. It is important that KNCS and C_6_H_12_N_4_, which are components of mixture inhibitors, are poor inhibitors by themselves. For example, introducing KNCS and C_6_H_12_N_4_ into a solution of phosphoric acid reduces the corrosion rate by only 2.1 and 1.5 times, respectively.

The high efficiency of triazole and composite inhibitors based on it in a 2 M H_3_PO_4_ solution is due to the fact that these compounds have a significant effect on the rates of partial processes of cathodic and anodic reactions occurring during LS corrosion.

### 3.5. Study of the Corrosion—Mechanical Characteristics of HS in the Presence of CorIs

The influence of CorIs on the plasticity of metal should be most pronounced on HS [24,25]. In accordance with the data of electrochemical studies, mixed inhibitors—TrzD + KNCS and TrzD + C_6_H_12_N_4_—not only significantly slow down the corrosion destruction of HS in a 2 M H_3_PO_4_ solution but also significantly slow down the process of hydrogen penetration into the metal volume. The hydrogen concentration in HS ($C_{H}^{v}$) determined by vacuum extraction decreases significantly in the presence of mixed inhibitors (Table 5). As a result, the HS iron alloy, which is prone to deterioration of mechanical properties upon hydrogen absorption, retains plasticity almost completely (*p* = 97 and 99%) after exposure to 2 M H_3_PO_4_ containing TrzD + KNCS or TrzD + C_6_H_12_N_4_. In the absence of the composite inhibitors, the decrease in the ductility of HS during its corrosion in a 2 M H_3_PO_4_ solution is very significant (*p* = 48%) (Table 5).

Thus, mixed TrzD-based CorIs feature a unique ability not only to slow down electrode reactions on the iron alloy but also to prevent the reduction in the volumetric sorption of hydrogen released during the cathodic reaction. This effect is extremely important in the protection of high-strength steel since not only the overall corrosion of the steel is hindered, but also its ductility is preserved. 

### 3.6. Adsorption Energy of TrzD on the LS Surface in H_3_PO_4_

The surface sorption energy (−Δ*G*_ads_) of a compound on a metal surface quantitatively characterizes the nature of its bond with the protected metal. If (−Δ*G*_ads_) > 40 kJ/mol, we can conclude that the adsorbed compound chemically reacts with the metal [45]. It is the chemical reaction of a CI with the metal surface that can provide efficient protection of the latter.

The surface sorption of TrzD on the LS in a 2 M H_3_PO_4_ solution, both in the absence and in the presence of 0.5 mmol/L KNCS, is described by the isotherm proposed by Temkin:

(17) $$\theta_{inh}=\frac{1}{f}ln\left[BC_{inh}\right]$$

where *θ_inh_* is the coverage with the TrzD, *f* is the coefficient of energy disparity of the surface, *B* is the sorption equilibrium constant, and *C*_inh_ is the content of CorI in an aggressive environment (Figure 5). In the absence of KNCS, the calculated value of parameter *f* is 7.31, and *B* is 1.76 × 10^7^ L/mol. In the presence of even a small amount of KNCS (0.5 mmol/L), the coefficient of energy disparity of the surface decreases to 6.29, but the sorption equilibrium constant increases to 2.38 × 10^7^ L/mol. The free energy of surface sorption, which is determined using the relationship

(18) $$B=\frac{1}{55.5}\exp\left[\frac{-\Delta G_{ads}}{RT}\right]$$

is (−Δ*G*_ads_) = 51 ± 1 kJ/mol. The obtained value of the surface sorption energy of TrzD on LS allows us to draw a conclusion about the chemical interaction between the LS surface and CorI molecules. When KNCS is introduced into an aggressive environment, the value of the free energy of surface sorption does not change (Table 6).

### 3.7. Layers Formed by TrzD on LS in H_3_PO_4_ Solution

**Figure 7:**
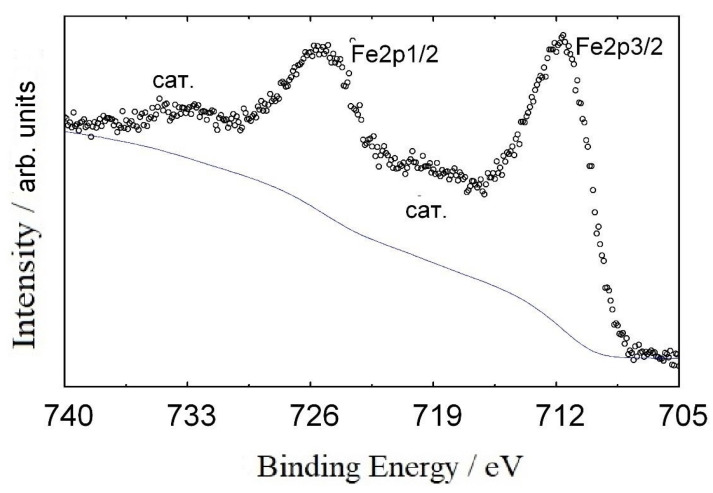
Spectrum of an LS coupon in the energy range of the Fe2*p*_3/2_ and Fe2*p*_1/2_ signals.

**Figure 8:**
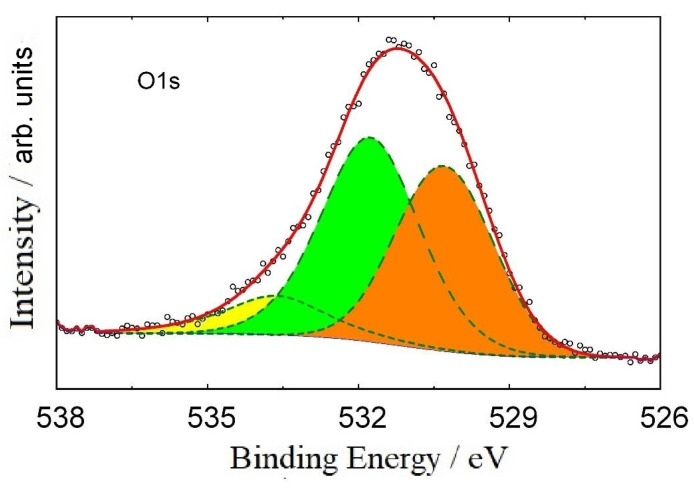
Spectrum of an LS coupon in the energy range of the O1*s* signals.

XPS spectroscopy is an efficient method for studying the structure of protective surface layers formed by organic CorIs on different surfaces. Based on the nature of the Fe2*p*_3/2_ and Fe2*p*_1/2_ signals (Figure 7), it can be assumed that the surface of LS kept for 24 h in 2M H_3_PO_4_ + TrzD + KNCS contains a layer consisting of Fe_3_O_4_ and FeOOH. We identified three types of oxygen atoms associated with oxygen atoms in the lattice of iron oxides (530.3 eV), with surface hydroxyl groups (531.8 eV) and surface sorbed water molecules (Eb = 533.5 eV) (Figure 8).

**Figure 9:**
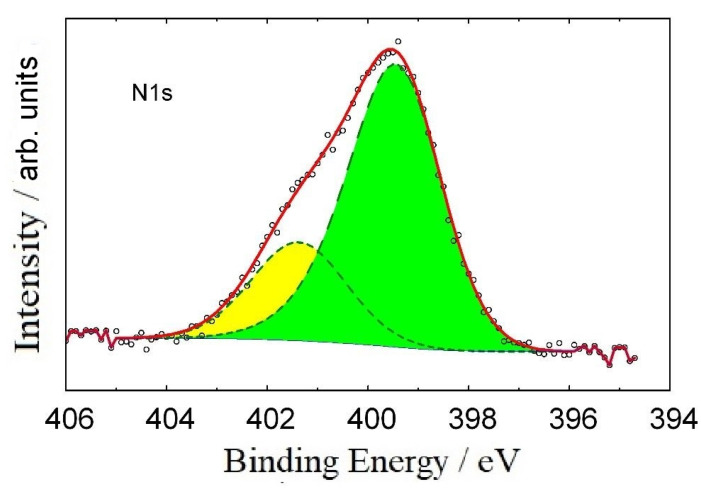
Spectrum of an LS coupon in the energy range of the N1*s* signals.

After thorough cleaning of the samples in an aqueous environment under the influence of ultrasound, a complex XPS spectrum of N1s electrons (Figure 9) indicates the presence of a CorI film on the surface of the drug, which was in contact with the mixture (H_3_PO_4_ + TrzD + KNCS) for 24 h. The observed spectrum can be decomposed into two peaks (399.5 and 401.4 eV) with an area ratio of 2:7. The second peak should be due to the presence of nitrogen atoms of the triazole ring. This peak, in the case of recording the S2p electronic peak, includes a signal from the nitrogen atoms of the thiocyanate group, the peak position of which practically coincides with the position of the peak of the nitrogen atoms of the triazole ring. In addition to the N1*s* and S2*p* peaks, the P2*p* peak (133.6 eV) associated with protonated phosphate anions is observed on the surface of samples kept in phosphoric acid.

**Scheme 1:**
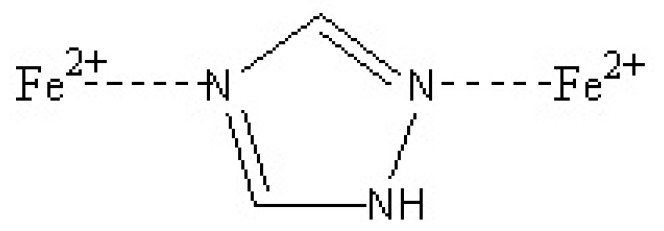
Fragment of the complex compound [Fe(Trz)_2_(NCS)_2_]_n_.

**Scheme 2:**
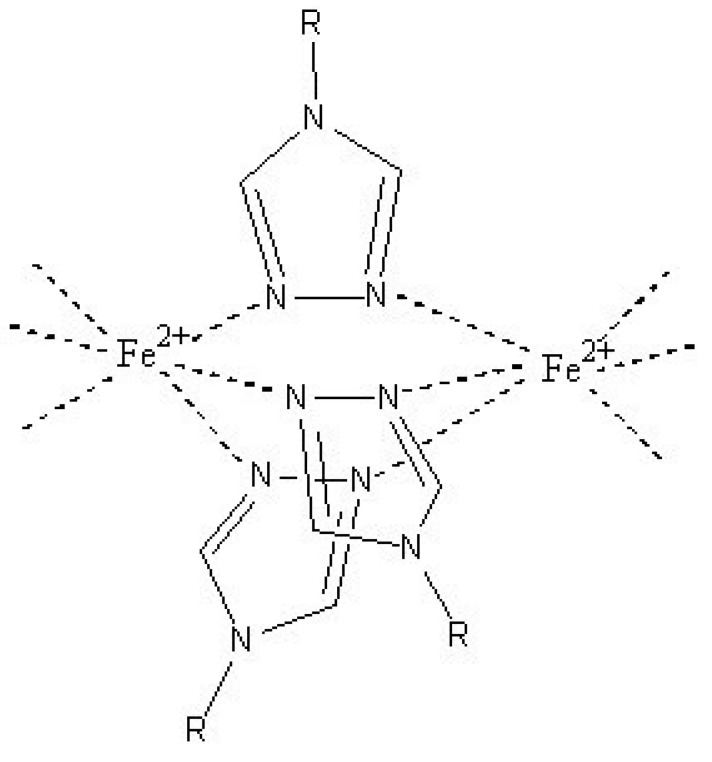
Fragment of the complex compound [Fe(Trz–C_18_H_37_)_3_]_n_.

We believe that the polymer structure of the protective inhibitor layer is formed due to the coordination reaction of the Fe(II) cations, which appear in the solution upon dissolution of the metal substrate, with the N atoms of the heterocycle of TrzD molecules and thiocyanate anions. Two types of Fe(II) complex compounds containing the substituted 1,2,4-triazole (Trz) and the thiocyanate anion are known: Fe(Trz)_4_(NCS)_2_ and Fe(Trz)_2_(NCS)_2_ [46,47,48,49]. Complex compounds of Fe(II) and substituted triazoles often have a polymer structure [47,50,51,52] determined by the bidentate nature of the heterocyclic ligand (Scheme 1 and Scheme 2). 

We believe that the mononuclear complex with the composition Fe(Trz)_4_(NCS)_2_ cannot provide a high protective effect if it forms a surface layer on the metal. Such a protective layer would be easily removed by washing, especially under ultrasonic conditions. In contrast, the complex with the composition Fe(Trz)_2_(NCS)_2_ with a polymeric structure is of interest. If such a complex is formed on LS, it can bind strongly to the metal substrate due to the lone pair of electrons of the N atoms of the heterocycle and the lone pair of electrons of the N and S atoms of the thiocyanate anion, which should provide effective protection. The bidentate nature of the rhodanion is manifested in the formation of some triazole complexes of d-metals [47,49], and the ability to be superficially sorbed on LS through terminal nitrogen or sulfur atoms is shown in [53].

A quantitative analysis of the ratio of atoms, obtained from a comparison of the areas of XPS spectra, suggests that a polymolecular layer more than 4 nm thick, including TrzD molecules, Fe(II) cations and thiocyanate anions, is formed on the LS surface within 24 h. After thoroughly washing the LS coupons with distilled water in an ultrasonic bath (18 min), a layer of CorI with a thickness of 3 ± 0.5 nm remains on the metal surface. This result indicates the formation of a polymer film on the iron alloy with a thickness of 3–4 conventional monolayers of CorI. The polymer complex has an approximate proportion of components: 1 TrzD molecule, 0.5 Fe atom and 0.4 ± 0.2 NCS^−^ or 1 TrzD molecule, 0.25 Fe atom and 0.4 ± 0.2 NCS^−^ (which structurally should be close to the complex of composition Fe(Trz)_4_(NCS)_2_). This inhibitor layer is chemisorbed on LS locally coated with Fe hydroxides and oxides.

**Figure 10:**
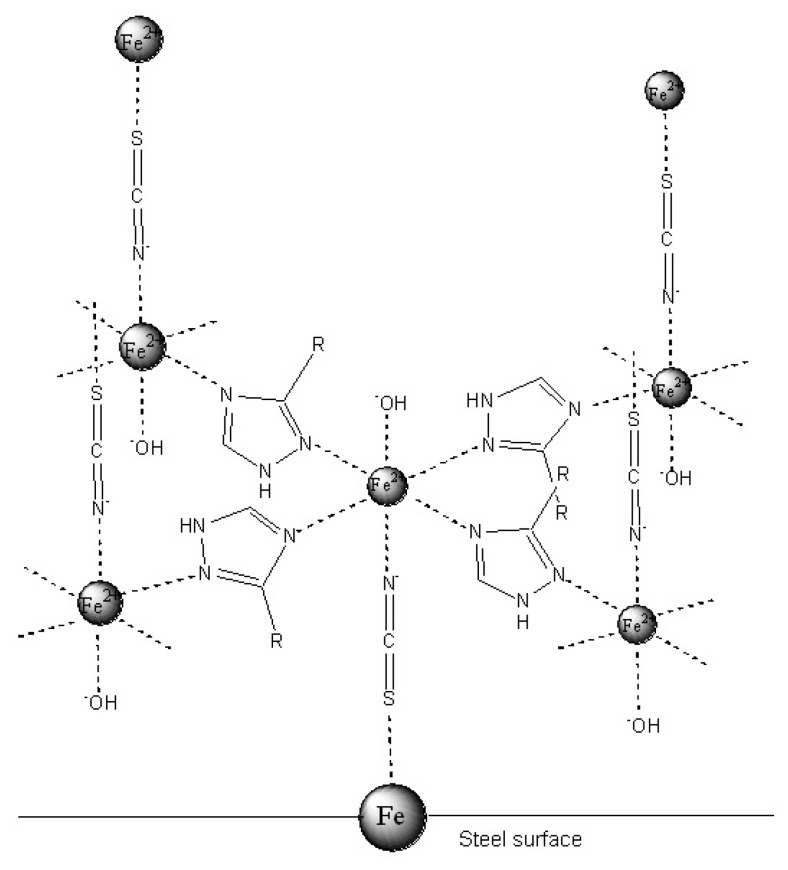
Assumed structure of the layer of the TrzD formed on LS in 2 M H_3_PO_4_ + TrzD + KNCS. *t* = 20 ± 1 °C.

The XPS spectrum of Fe2*p* electrons does not provide information sufficient for distinguishing the component due to iron atoms that form any of the possible complexes. We hypothesize that the protective layer formed on the iron alloy contains a complex with the component ratio of 1 TrzD molecule, 0.5 Fe atom and 0.4 ± 0.2 NCS^−^, since the mononuclear complex with the composition Fe(Trz)_4_(NCS)_2_, should it form surface layers on LS, cannot provide efficient protection. The structure of the resulting protective layer should be similar to that of polynuclear complexes with the composition Fe(Trz)_2_(NCS)_2_, where the triazole molecules are linked by nitrogen heteroatoms in positions 1 and 4 with bridging Fe(II) cations into polymer layers (Figure 10). These layers are bound with each other and with the surface iron atoms by bidentate thiocyanate anions, which react with the surface metal atoms and Fe(II) cations. It is this structure that matches most closely the film composition that we determined using XPS spectral data. However, it is possible that other variants of the structure of the inhibitor protective film can be formed.

When samples are washed ultrasonically, the less structured and more diffused upper layers of the CorIs, which are more weakly bound with the underlying layers, are removed. However, the chemical reaction within the more structured molecular layers of the T3D inhibitor, directly adjacent to the metal, is sufficient to retain them on the surface of the LS during thorough washing and during XPS examinations under high-vacuum conditions. It should be noted that the electron spectra of Fe2p show that the metal surface under the CorI layer contains oxide Fe(III). We believe that the oxide forms on the LS when the samples are washed in distilled water and dried. In addition, the oxide phase contains a certain proportion of acid phosphates. This is indicated by the presence of a P2p electronic peak in the spectrum of the LS coupon.

### 3.8. Atomic Force Microscopy

Visual surface inspection and measurements of the surface potential also provide vast additional information about the inhibitor’s capabilities. Micrographs, topographic maps and surface potential distribution of an LS sample before and after etching in 2 M phosphoric acid with various inhibitor formulations are presented in Table 7.

The micrograph and topographic map of the LS sample show defects due to surface cleaning. The average roughness of such a surface is 46.7 nm. Etching in 2 M H_3_PO_4_ leads to the emergence of a clearly visible layer of sludge on the LS, while the average surface roughness increases to 392 nm.

The introduction of TZD into a corrosive environment also leads to the emergence of an uneven layer of sludge on the LS. The micrograph displays areas without a visible sludge layer; they were used to obtain topographic maps and potential distribution. They showed that the surface roughness increased to 163 nm compared to the background value.

The use of two- and three-component composite inhibitors leads to the deposition of needle-shaped crystals on the LS. In the case of the two-component mixture (TrzD + KNCS), they are larger, their length reaches 80 µm, and in the case of the three-component formulation (TrzD + KNCS + C_6_H_12_N_4_), they are 30 µm long. Both formulations preserve the metal surface considerably: the average roughness is 114 nm and 112 nm, respectively, for the two- and three-component formulations.

The addition of potassium thiocyanate to phosphoric acid leads to the appearance of a visible translucent layer with complex topography on the surface, while the surface roughness increases to 297 nm.

The addition of hexamethylenetetramine resulted in the appearance of a thick layer of sludge on the LS, while the surface roughness reached the maximum value among all the samples, 431 nm.

Thus, minimal damage to the LS surface in 2 M H_3_PO_4_ solutions is observed in media containing two- and three-component inhibitors. This result agrees with the data on the corrosion of samples obtained by weight measurements.

### 3.9. Kelvin Probe Force Microscopy

The surface potential distributions of the steel sample before and after etching in phosphoric acid solution with different inhibitor compositions are presented in Table 8.

The surface potential map of the initial surface of the metal sample is quite homogeneous; its average value is 0.44 V. Its etching in phosphoric acid for 2 h reduces the value of the surface potential to 0.29 V. Moreover, the distribution of the potential on the surface is quite uneven, which suggests inhomogeneous etching of the surface and different thicknesses of the sludge layer. The decrease in surface potential is probably also connected with the phosphatization of the surface. Soaking the metal sample in phosphoric acid with TrzD + KNCS and TrzD + KNCS + C_6_H_12_N_4_ additives reduces the surface potential of the metal to 0.21V and 0.07V, respectively. Also, Table 8 shows the values of electron yield work for LS before and after etching in H_3_PO_4_. The electron yield work increases in the order of “Zero” surface ˂ H_3_PO_4_ ˂ TrzD ˂ TrzD + KCNS ˂ TrzD + KCNS + C_6_H_12_N_4_, which for composite inhibitors correlates with the data of their protective effect obtained during corrosion studies. Thin protective layers formed on the LS surface change the properties of the metal surface, increasing the work of electron yield.

## 4. Conclusions

1. The addition of a TrzD inhibitor and compositions based on it slows down the cathodic reaction on iron alloys in a H_3_PO_4_ solution and inhibits the surface and volumetric sorption of H atoms by the metal. The use of (IPZ) made it possible to calculate the kinetic parameters of HER of its main stages in an aggressive environment containing inhibitory compositions. In the presence of CorIs, the reaction rate of the discharge of H^+^ ions and the volumetric sorption of hydrogen by iron alloys significantly slow down. The addition of 5 mM TrzD + 0.5 mM KNCS and 5 mM TrzD + 0.5 mM KNCS + 200 mM C_6_H_12_N_4_ reduces the concentration of diffusion-mobile hydrogen in LS by 11 and 5.8 times, respectively.
2. At the corrosion potential, the 5 mM TrzD + 0.5 mM KNCS and 5 mM TrzD + 0.5 mM KNCS + 200 mM C_6_H_12_N_4_ inhibitors decrease the total hydrogen concentration in HS up to 9.3- and 11-fold at 20 ± 1 °C (the degree of HS protection from hydrogen sorption is 89 and 91%). As a result of reducing the hydrogen content in the metal volume with the TrzD + KNCS and TrzD + KNCS + C_6_H_12_N_4_ formulations, its plastic properties virtually do not change during corrosion in H_3_PO_4_ solutions, while its resistance to cracking increases significantly.
3. The TrzD + KNCS and TrzD + KNCS + C_6_H_12_N_4_ significantly reduce the rate of the anodic process of LS in a H_3_PO_4_ solution. This phenomenon, combined with the deceleration of the rate of the cathodic evolution of hydrogen, determines the efficiency of these mixtures as CorIs of iron alloys. The addition of 5 mM TrzD + 0.5 mM KNCS and 5 mM TrzD + 0.5 mM KNCS + 200 mM C_6_H_12_N_4_ reduces LS corrosion in 2 M H_3_PO_4_ (*t* = 20 ± 1 °C) by almost 33- and 51-fold.
4. The efficient slowdown of the iron alloy corrosion TrzD-based mixtures and the preservation of the ductility of the metal are conditioned by the specific features of the mechanism of its inhibitory action. Protection of iron alloys in H_3_PO_4_ solutions by this compound is due to the formation of a layer of a polymer complex compound essentially consisting of triazole molecules on the metal. The layer of the polymer complex compound directly adjacent to the metal is chemically bound to it.

## Figures and Tables

**Table 1:** Protection of steels in H_3_PO_4_ solutions with various corrosion inhibitors.

Inhibitor	Corrosive Environment	Steel	*Z*, %	Ref.
10 mM Benzyltrimethyl-ammonium iodide	7 M H_3_PO_4_ (20 °C)	Cold rolled steel	79.8	[12]
1 mM Methyl Violet + 50 mM NaBr	1 M H_3_PO_4_ (25–40 °C)	Mild steel	91.8–92.2	[13]
2.5 mM Brilliant Green oxalate + 0.5 mM KNCS	2 M H_3_PO_4_ (25–95 °C)	St3 mild steel	91.3–96.5	[14]
0.1 mM 2-Mercaptothiazoline	1–9 M H_3_PO_4_ (30 °C)	A1010 grade low-carbon steel	82.0–92.0	[15]
2.0 mM sodium oleate + 2.0 mM Ce(SO_4_)_2_	3 M H_3_PO_4_ (20 °C)	Cold rolled steel	97.5	[16]

**Table 2:** Corrosion potentials (*E*_cor_) of LS in 2 M H_3_PO_4_, cathodic and anodic current densities (*i*_c_ and *i*_a_) and inhibitory factor of the cathodic and anodic reactions (*Z*_c_ and *Z*_a_) at *E* = −0.30 and −0.20 V, respectively. *t* = 20 ± 1 °C.

Additive	−*E*_cor_, V	*i*_c_, A/cm^2^	*Z*_c_, %	*i*_a_, A/cm^2^	*Z*_a_, %
None (background)	0.28	2.8 × 10^−4^	-	5.3 × 10^−3^	-
TrzD	0.26	1.2 × 10^−5^	95.7	3.2 × 10^−5^	99.4
TrzD + KNCS	0.26	8.4 × 10^−6^	97.0	4.5 × 10^−5^	99.2
TrzD + KNCS + C_6_H_12_N_4_	0.27	1.2 × 10^−5^	95.7	6.7 × 10^−5^	98.7
KNCS	0.28	7.3 × 10^−5^	73.9	1.3 × 10^−3^	75.5
C_6_H_12_N_4_	0.27	3.5 × 10^−4^	−25.0	2.1 × 10^−3^	60.4

**Table 3:** The kinetic parameters of HER on LS in 2 M H_3_PO_4_. *t* = 20 ± 1 °C.

Additive	*k*_1,i_, mol/(cm^2^ s)	*k*, cm^3^/mol	*k*_r_, mol/(cm^2^ s)	*θ*_H_ ^∗^ × 100	$C_{H}^{s}$, ^∗^ mol/cm^3^
None (background)	3.21 × 10^−9^	3.75 × 10^5^	1.0 × 10^−5^	1.63	5.8 × 10^−8^
TrzD	1.24 × 10^−10^	3.73 × 10^6^	1.03 × 10^−7^	1.52	7.0 × 10^−9^
TrzD + KNCS	8.70 × 10^−11^	9.31 × 10^5^	6.59 × 10^−7^	0.45	5.1 × 10^−9^
TrzD + KNCS + C_6_H_12_N_4_	1.24 × 10^−10^	6.21 × 10^6^	1.8 × 10^−8^	0.95	1.0 × 10^−8^

^∗^ Calculated values are given for LS potential −0.3 V.

**Table 4:** Effect of inhibitors and their mixtures on the corrosion process of LS in 2 M H_3_PO_4_. *t* = 20 ± 1 °C.

Additive	*ρ*, g/(m^2^ h)	*CR*, mm/Year	*Z*_cor_, %
None (background)	7.8	8.7	-
TrzD	1.8	2.0	77
TrzD + KNCS	0.23	0.26	97
TrzD + KNCS + C_6_H_12_N_4_	0.15	0.17	98
KNCS	3.7	4.2	53
C_6_H_12_N_4_	5.2	5.8	33

**Table 5:** Effect of inhibitors and their mixtures on the corrosion and mechanical properties of HS in 2 M H_3_PO_4_. *t* = 20 ± 1 °C.

Additive	*ρ*, g/(m^2^ h)	*CR*, mm/Year	*Z_cor_*, %	$C_{H}^{v}$*,* mol/cm^3^	*Z^v^_H_*, %	*p*, %
Background	16	18	-	2.7 × 10^−5^	-	48
TrzD + KNCS	0.25	0.28	98	2.9 × 10^−6^	89	97
TrzD + KNCS + C_6_H_12_N_4_	0.22	0.25	99	2.5 × 10^−6^	91	99

**Table 6:** Parameters of the Temkin isotherm for the surface sorption of TrzD on LS in H_3_PO_4_ solution. *t* = 20 ± 1 °C.

Additive	*f*	*B*, L/mol	(−Δ*G*_ads_), kJ/mol
2 M H_3_PO_4_	7.31	1.76 × 10^7^	51 ± 1
2 M H_3_PO_4_ + 0.5 mmol/L KNCS	6.29	2.38 × 10^7^	51 ± 1

**Table 7:** Results of visual inspection along with Kelvin probe force microscopy of an LS electrode before and after treatment in 2 M H_3_PO_4_ (*t* = 20 ± 1 °C) with and without various inhibitory formulations.

Conditions	Micrograph 400 × 600 μm	Topographic Map 25 × 25 μm
“Zero” surface	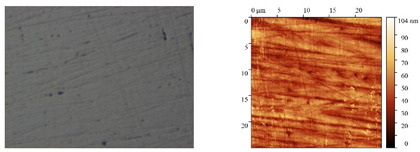	
Background	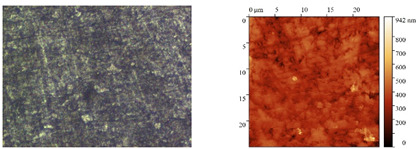	
TrzD	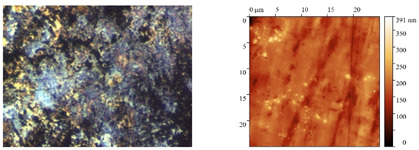	
TrzD + KNCS	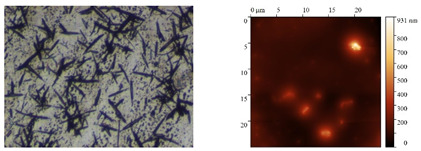	
TrzD + KNCS + C_6_H_12_N_4_	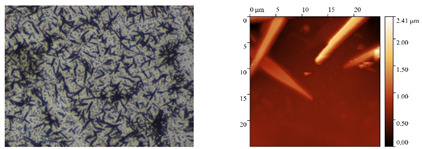	
KNCS	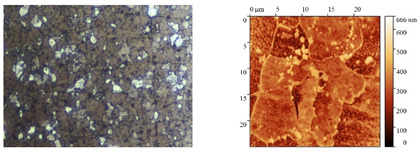	
C_6_H_12_N_4_	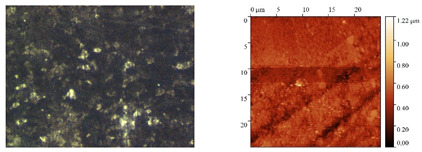	

**Table 8:** Surface potential distribution maps of steel samples before and after treatment in 2 M H_3_PO_4_ without and with different inhibiting compositions and average value of electron yield work.

Conditions	Surface Potential Distribution Map	VCPD, eV
“Zero” surface	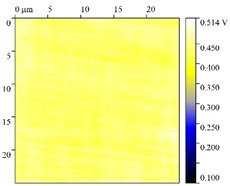	4.36
Background	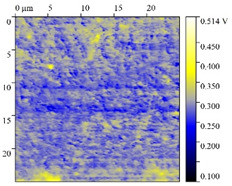	4.51
TrzD	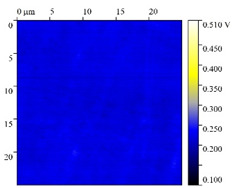	4.58
TrzD + KNCS	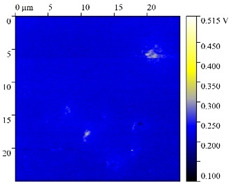	4.59
TrzD + KNCS + C_6_H_12_N_4_	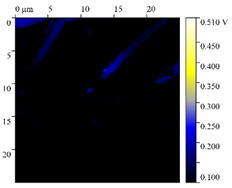	4.73

## Data Availability

The data presented in this study are available on request from the corresponding author.
